# Antithrombotic Therapy for Chronic Kidney Disease Patients With Concomitant Atrial Fibrillation and Coronary Artery Disease

**DOI:** 10.3389/fcvm.2021.751359

**Published:** 2021-10-08

**Authors:** Kuo-Hua Lee, Shuo-Ming Ou, Yuan-Chia Chu, Yao-Ping Lin, Ming-Tsun Tsai, Der-Cherng Tarng

**Affiliations:** ^1^Division of Nephrology, Department of Medicine, Taipei Veterans General Hospital, Taipei City, Taiwan; ^2^Faculty of Medicine, School of Medicine, National Yang Ming Chiao Tung University, Taipei, Taiwan; ^3^Institute of Clinical Medicine, National Yang Ming Chiao Tung University, Taipei City, Taiwan; ^4^Center for Intelligent Drug Systems and Smart Bio-devices (IDS^2^B), Hsinchu, Taiwan; ^5^Information Management Office, Taipei Veterans General Hospital, Taipei City, Taiwan; ^6^Big Data Center, Taipei Veterans General Hospital, Taipei City, Taiwan; ^7^Department and Institute of Physiology, National Yang-Ming University, Taipei City, Taiwan

**Keywords:** anticoagulation, acute myocardial infarction, atrial fibrillation, stroke, thromboembolism

## Abstract

**Background:** Oral anticoagulants (OAC) plus antiplatelets is recommended for patients with atrial fibrillation (AF) and coronary artery disease (CAD) to reduce thromboembolism. However, there is limited evidence regarding antithrombotic therapy for patients with concomitant chronic kidney disease (CKD), AF, and CAD, especially those not undergoing percutaneous coronary intervention. We aimed to use real-world data assessing the efficacy and safety of antithrombotic regimens in this population.

**Methods:** We used a single-center database of 142,624 CKD patients to identify those receiving antithrombotic therapy for AF and CAD between 2010 and 2018. Patients taking warfarin or direct OAC (DOAC) alone were grouped in the OAC monotherapy (*n* = 537), whereas those taking OAC plus antiplatelets were grouped in the combination therapy (*n* = 2,391). We conducted propensity score matching to balance baseline covariates. The endpoints were all-cause mortality, major adverse cardiovascular events, and major bleedings.

**Results:** After 1:4 matching, the number of patients in OAC monotherapy and combination therapy were 413 and 1,652, respectively. Between the two groups, combination therapy was associated with higher risks for ischemic stroke (HR 2.37, CI 1.72–3.27), acute myocardial infarction (HR 6.14, CI 2.51–15.0), and hemorrhagic stroke (HR 3.57, CI 1.35–9.81). The results were consistent across CKD stages. In monotherapy, DOAC users were associated with lower risks for all-cause mortality, AMI, and gastrointestinal bleeding than warfarin, but the stroke risk was similar between the two subgroups.

**Conclusions:** For patients with concomitant CKD, AF and CAD not undergoing PCI, OAC monotherapy may reduce stroke and AMI risks. DOAC showed more favorable outcomes than warfarin.

## Introduction

Patients with chronic kidney disease (CKD) have a high risk of cardiovascular (CV) comorbidities including atrial fibrillation (AF) and coronary artery disease (CAD). These three disease entities concomitantly affect each other and share common risk factors such as age, smoking, hypertension, dyslipidemia, and diabetes mellitus (DM). Approximately 5–10% of patients with CKD concomitantly have AF and CAD, and the coexistence creates a vicious cycle (1). With CKD progression, the loss of antioxidant capacity enhances the progression of coronary atherosclerosis and vascular calcification that aggravate myocardial ischemia. The increasing severity of CAD and subsequent cardiac remodeling might predispose individuals to AF. In addition, AF can reduce cardiac output, thus accelerating the deterioration of CKD. Consequently, these patients experience rapid deterioration in renal function and develop adverse CV events.

Combined oral anticoagulants (OAC) and antiplatelets have been suggested for antithrombotic therapy in patients with both AF and CAD. However, such a combination may lead to adverse effects such as hemorrhagic stroke and major bleeding, especially in patients with CKD. Accordingly, previous research aimed to investigate modified antithrombotic regimens with better efficacy and safety for these high-risk patients. Previous randomized controlled trials (RCT) have shown that the combination of warfarin plus clopidogrel exerted an antithrombotic effect equal to that exerted by the conventional triple therapy of warfarin, aspirin, and clopidogrel on patients with AF undergoing percutaneous coronary intervention (2, 3). In comparison to triple therapy, the RE-DUAL trial reported that the use of dabigatran plus clopidogrel leads to comparable CV outcomes and causes less bleeding (4). Furthermore, Yasuda et al. demonstrated that rivaroxaban monotherapy was associated with a lower risk of major bleeding and was non-inferior to rivaroxaban combined with a single antiplatelet agent in terms of efficacy in patients with AF and stable CAD undergoing PCI (5). However, because the number of patients with CKD [defined as those with an estimated glomerular filtration rate (eGFR) of <60 mL/min per 1.73 m^2^] in these RCTs was low, evidence regarding the safety of antithrombotic therapy in patients with CKD remains limited. Moreover, PCI in the management of CAD among patients with CKD is sometimes limited to the risk of contrast-induced renal failure. Therefore, in this study, we investigated the efficacy and safety of antithrombotic therapy in patients with CKD with concomitant AF and CAD in the Evaluating the Prognosis and Impacts in CKD (EPIC) Research of Taipei Veterans General Hospital (VGH).

## Methods

### Data Source

This study was based on a single-center, retrospective, observational design. Our main data source was derived from the Big Data Center (BDC) of Taipei VGH. The database contains data regarding demographic characteristics, diagnostic codes, imaging studies, medical procedures, and laboratory findings for outpatient appointments, emergency department visits, and inpatient admissions from January 2010 through December 2018. We used codes from the International Classification of Diseases, Ninth and Tenth Revision (ICD-9 and ICD-10, respectively) to screen the diagnosis of CKD (ICD-9: 585 and ICD-10: N18), non-valvular AF (ICD-9: 427.31 and ICD-10: I48), and CAD (ICD-9: 410, 411, 412, 413, and 414 and ICD-10: I20, I21, I22, I23, I24, and I25). In addition, we used electronic medical record (EMR) system to collect data that were not included or completely recorded in the Taipei VGH BDC, such as social history, event records, and drug prescriptions. The study protocol was approved by the Institutional Review Board of the Taipei VGH (2017-09-002BC) and fulfilled the ethical guidelines of the Declaration of Helsinki.

### Participants

We enrolled patients who had concurrent CKD, CAD, and AF indicated for OAC therapy such as warfarin or direct OACs (DOACs, referred to as apixaban, dabigatran, rivaroxaban, and edoxaban). Patients who met all the following criteria were considered to be eligible for participation: (1) age >20 years, (2) males with a baseline CHA_2_DS_2_-VASc score of >2 and females with a baseline CHA_2_DS_2_-VASc score of >3, and (3) use of OACs. Exclusion criteria were as follows: (1) history of PCI before enrollment, (2) use antiplatelets other than aspirin and clopidogrel, (3) use OACs <90 days, (4) no availability of serum creatinine and urine protein measurements at baseline and follow-up. According to the prescriptions, patients receiving warfarin or DOACs alone were grouped into the OAC monotherapy group, whereas patients receiving an OAC plus antiplatelets were grouped into the combination therapy group.

### Follow-Up and Endpoints

The index date was defined as the first prescription of OACs. Patients were followed up since the index date until death, loss to follow-up, censoring, or December 31, 2018. We included laboratory tests associated with CV risks such as total and low-density lipoprotein cholesterol, triglyceride, glucose, glycated hemoglobin, and hemoglobin. In addition, the baseline serum creatinine and urine protein-to-creatinine ratio were collected. Data of these measurements closest to the index date within 1 month were defined as the baseline. The eGFR values were calculated for serum creatinine by using the Chronic Kidney Disease Epidemiology Collaboration equation. We identified concomitant medications by using the EMR, and only drug exposure within 90 days before the index date was included. Comorbidity patterns in this study were hypertension, DM, congestive heart failure (CHF), and malignancy. Primary outcomes were all-cause mortality and major adverse cardiac events (MACE) including ischemic stroke, acute myocardial infarction, transient ischemic attack, peripheral artery occlusive disease, and hospitalization for CHF. Secondary endpoints were major bleeding, including hemorrhagic stroke, gastrointestinal (GI) bleeding, and other bleeding events. Renal outcomes included CKD progression characterized by the first occurrence of eGFR declines of >20, >30, >40, and >50%, end-stage renal disease (ESRD), and initiation of dialysis.

### Statistical Analysis

Missing values were imputed using the multiple imputation method by fully conditional specification with five repetitions to establish a complete dataset. The baseline characteristics were compared between the two groups of patients by using the *x*^2^ test for categorical variables and independent *t*-test and Mann–Whitney *U*-test for parametric and non-parametric continuous variables, respectively. Propensity scores were calculated with all baseline covariates by using a logistic regression model, and propensity score matching was conducted through the nearest neighbor approach with a caliper of 0.01. The standardized difference was calculated to assess the balance between the two groups after matching, and a difference of <0.2 in the score was considered to indicate a negligible imbalance.

We used the as-treated approach to account for switching antithrombotic medications in a real-world setting. The treatment effect for the time to the first event was estimated using Cox proportional-hazards models. The strength of the association between the exposure and outcome is presented as the hazard ratio (HR) with the 95% confidence interval (CI). The cumulative incidences of all-cause mortality, adverse CV events, CKD progression, and major bleeding were compared among patients receiving different antithrombotic therapies by using the modified Kaplan–Meier method and tested using the log-rank statistic. A *P* < 0.05 was considered statistically significant. All analyses were conducted using SAS 9.4 software (SAS Institute Inc., Cary, NC, USA).

## Results

### Study Population Characteristics

**Figure 1:**
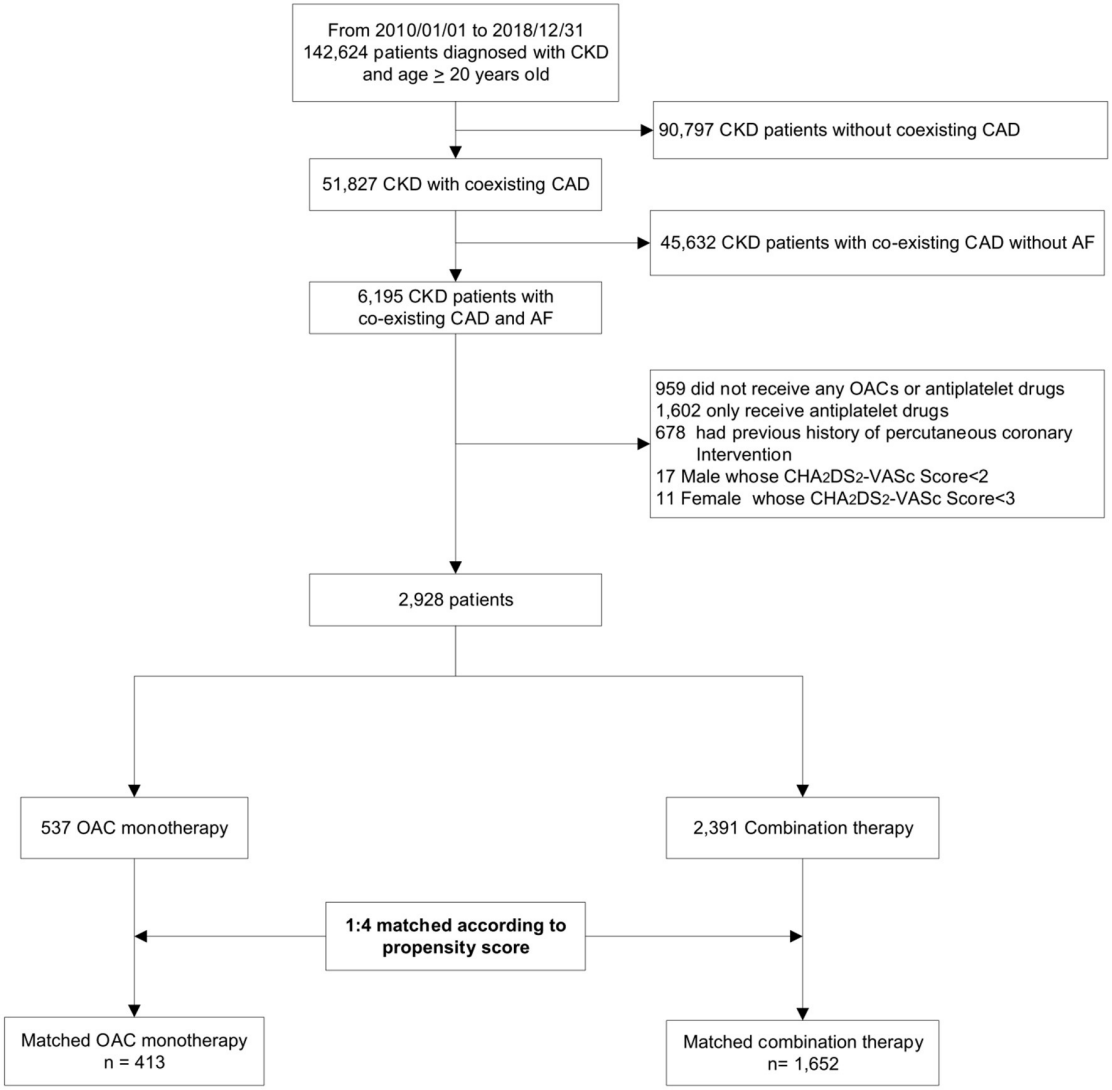
Flowchart of study enrollment. This hospital-based cohort included a total of 142,624 patients with CKD between 2010 and 2018. After excluding ineligible patients, we identified 2,928 patients with concurrent AF and CAD not undergoing percutaneous coronary intervention, and divided them into OAC monotherapy group (*n* = 537) and combination therapy (OAC plus antiplatelets, *n* = 2,391) according to their antithrombotic regimens. Finally, we conducted a 1:4 propensity-score matching to balance baseline covariates. CKD, chronic kidney disease; CAD, coronary artery disease; AF, atrial fibrillation; OAC, oral anticoagulant.

**Figure 2:**
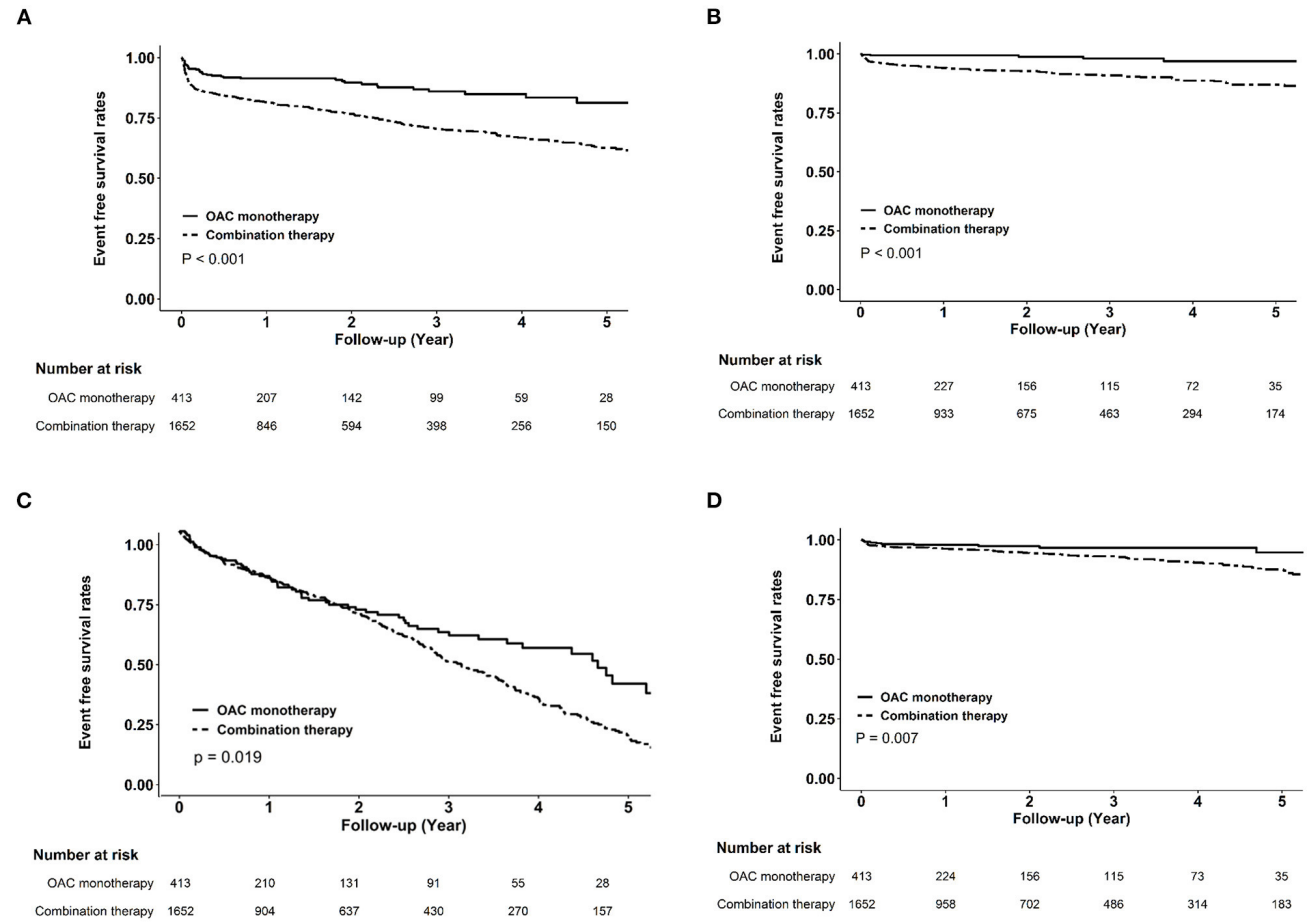
Kaplan–Meier curves of clinical endpoints between oral anticoagulant monotherapy and combination therapy in the matched cohort. Compared with combination therapy, oral anticoagulant monotherapy was associated with higher cumulative event-free probabilities for ischemic stroke **(A)**, myocardial infarction **(B)**, all-cause mortality **(C)**, and hemorrhagic stroke **(D)**.

The patient enrollment process is depicted in Figure 1. A total of 142,624 patients were diagnosed of CKD between 2010 and 2018. After excluding patients who did not meet the inclusion criteria, we identified 2,928 patients with concomitant CAD and AF indicated for OAC therapy stratified by the CHA_2_DS_2_-VASc score. Among eligible participants, 537 were included into the OAC monotherapy group, whereas 2,391 were included into the combination therapy group. The baseline clinical characteristics of these patients are listed in Table 1. After 1:4 propensity score matching, the baseline covariates were comparable between the two groups. Furthermore, the distribution balance for the propensity score and the balance plot of absolute standardized effect sizes before and after matching are shown in Supplementary Table 1; Figures 1, 2, respectively.

**Table 1:** Baseline characteristics of the study population.

	**Before propensity score matching**			**After propensity score matching**		
	**OAC monotherapy**	Combination therapy^*^	**SMD**	**OAC monotherapy**	Combination therapy^*^	**SMD**
	**(*n* = 537)**	**(*n* = 2,391)**		**(*n* = 413)**	**(*n* = 1,652)**	
Age, years	78.5 [68.8, 84.2]	80.0 [71.2, 85.8]	0.143	79.8 [71.2, 84.8]	80.4 [72.0, 85.7]	0.041
Male, n (%)	338 (62.9)	1,697 (71.0)	0.171	298 (72.2)	1,171 (70.9)	0.028
Cholesterol, mg/dL	161.0 [139.0, 186.0]	160.0 [137.0, 183.0]	0.085	160.0 [138.0, 184.0]	161.0 [137.0, 183.0]	0.062
LDL, mg/dL	93.0 [77.0, 114.0]	92.0 [74.0, 113.0]	0.068	91.0 [76.0, 113.0]	92.0 [75.0, 113.0]	0.063
TG, mg/dL	92.0 [65.0, 128.0]	91.0 [67.0, 127.5]	0.061	92.0 [65.0, 127.0]	91.0 [67.0, 129.0]	0.065
Glucose, mg/dL	118.0 [101.0, 155.0]	118.0 [100.0, 150.0]	0.021	118.0 [102.0, 155.0]	117.0 [99.0, 150.0]	0.038
HbA1c, %	6.6 [5.9, 7.7]	6.7 [6.0, 8.0]	0.023	6.6 [6.0, 7.7]	6.6 [6.0, 7.8]	0.025
Hemoglobin, g/dL	12.7 [11.2, 13.9]	12.6 [11.2, 14.0]	0.011	12.8 [11.3, 14.0]	12.6 [11.2, 14.0]	0.023
**eGFR, mL/min/1.73 m** ^**2**^			0.121			0.088
>90	37 (6.9)	124 (5.2)		22 (5.3)	90 (5.4)	
60–89	209 (38.9)	849 (35.5)		164 (39.7)	592 (35.8)	
30–59	223 (41.5)	1,050 (43.9)		172 (41.6)	720 (43.6)	
15–29	44 (8.2)	240 (10.0)		37 (9.0)	175 (10.6)	
<15	24 (4.5)	128 (5.4)		18 (4.4)	75 (4.5)	
UPCR, mg/mg	0.2 [0.1, 0.9]	0.2 [0.1, 1.0]	0.050	0.2 [0.1, 1.0]	0.2 [0.1, 0.9]	0.032
Hypertension, *n* (%)	343 (63.9)	1,768 (73.9)	0.219	300 (72.6)	1,218 (73.7)	0.025
DM, *n* (%)	178 (33.1)	921 (38.5)	0.112	149 (36.1)	623 (37.7)	0.034
CHF, *n* (%)	247 (46.0)	1,068 (44.7)	0.027	179 (43.3)	732 (44.3)	0.02
Malignancy, *n* (%)	120 (22.3)	615 (25.7)	0.079	98 (23.7)	425 (25.7)	0.046
ACEIs/ARBs, *n* (%)	339 (63.1)	1,706 (71.4)	0.176	275 (66.6)	1,157 (70.0)	0.074
β-blockers, *n* (%)	305 (56.8)	1,545 (64.6)	0.161	264 (63.9)	1,039 (62.9)	0.021
α-blockers, *n* (%)	144 (26.8)	886 (37.1)	0.221	141 (34.1)	545 (33.0)	0.024
CCBs, *n* (%)	229 (42.6)	1,177 (49.2)	0.132	190 (46.0)	790 (47.8)	0.036
Statins, *n* (%)	126 (23.5)	911 (38.1)	0.321	126 (30.5)	504 (30.5)	<0.001
OHAs, *n* (%)	91 (16.9)	513 (21.5)	0.115	76 (18.4)	339 (20.5)	0.054
Insulins, *n* (%)	113 (21.0)	642 (26.9)	0.136	95 (23.0)	429 (26.0)	0.069

*Data are presented as n (%) or median [interquartile range]*.

* *Refers to oral anticoagulants plus antiplatelets*.

*OAC, oral anticoagulant; SMD, standardized mean difference; LDL, low-density lipoprotein; TG, triglyceride; HbA1C, glycated hemoglobin; eGFR, estimated glomerular filtration rate; UPCR, urine protein-to-creatinine ratio; DM, diabetes mellitus; CHF, congestive heart failure; ACEI, angiotensin converting enzyme inhibitor; ARB, angiotensin receptor blocker; CCB, calcium channel blockers; OHA, oral hypoglycemic agent*.

### MACE and Bleeding Risks Associated Wih OAC in Combination With Antiplatelets

Figure 2 shows the cumulative event-free probability curve of all-cause mortality, AMI, ischemic stroke, and hemorrhagic stroke between the two groups. During the follow-up period of 88.5 ± 66.4 months, patients receiving OAC monotherapy were found to have more favorable outcomes (all *P* < 0.05; log-rank test). As shown in Table 2, compared with the OAC monotherapy group, the combination therapy group had increased risks of all-cause mortality (HR 1.31, 95% CI 1.01–1.71, *P* = 0.044), ischemic stroke (HR 2.37, 95% CI 1.72–3.27, *P* < 0.001), and AMI (HR 6.14, 95% CI 2.51–15.0, *P* < 0.001). Moreover, the combination therapy group had a higher risk of hemorrhagic stroke (HR 3.57, 95% CI 1.3–9.81, *P* = 0.014). The risks of decline in eGFR and ESRD were similar between the two groups.

**Table 2:** Risks of all-cause mortality, progression of chronic kidney disease, adverse cardiovascular events and bleeding complications between oral anticoagulant monotherapy or combination therapy in patients with chronic kidney disease with atrial fibrillation and coronary artery disease.

**Outcomes**	**OAC monotherapy**	Combination therapy^*^	
	**HR (95% CI)**	**HR (95% CI)**	***P*-value**
**All-cause mortality**	Reference	1.31 (1.01–1.71)	0.044
**Progression of CKD**			
eGFR decline > 20%	Reference	0.88 (0.66–1.17)	0.383
eGFR decline > 30%	Reference	1.15 (0.75–1.77)	0.527
eGFR decline > 40%	Reference	1.08 (0.62–1.89)	0.788
eGFR decline > 50%	Reference	0.86 (0.39–1.89)	0.705
End-stage renal disease^†^	Reference	1.77 (0.62–5.04)	0.284
Composite renal outcomes^‡^	Reference	0.75 (0.30–1.88)	0.543
**Major adverse cardiac events**		
Ischemic stroke	Reference	2.37 (1.72–3.27)	<0.001
AMI	Reference	6.14 (2.51–15.0)	<0.001
Systemic embolism	Reference	1.17 (0.73–1.87)	0.511
TIA	Reference	1.33 (0.7–2.52)	0.386
PAOD	Reference	0.56 (0.22–1.44)	0.229
Hospitalization for CHF	Reference	0.99 (0.86–1.14)	0.906
**Bleeding complications**			
Hemorrhagic stroke	Reference	3.57 (1.35–9.81)	0.014
GI bleeding	Reference	1.14 (0.84–1.56)	0.405
Other bleeding	Reference	0.99 (0.75–1.31)	0.927

* *Refers to an oral anticoagulant plus antiplatelets*.

† *eGFR <15 mL/min per 1.73 m^2^ necessitating long-term dialysis*.

‡ *eGFR decline of 50% from baseline or eGFR <15 mL/min per 1.73 m^2^ necessitating long-term dialysis*.

*OAC, oral anticoagulant; HR, hazard ratio; CI, confidence interval; CKD, chronic kidney disease; eGFR, estimated glomerular filtration rates; ESRD, end-stage renal disease; AMI, acute myocardial infarction; TIA, transient ischemic attack; PAOD, peripheral artery occlusive disease; CHF, congestive heart failure; GI, gastrointestinal*.

### Subgroup Analyses of MACE and Bleeding Risks Associated With OAC in Combination With Antiplatelets

Supplementary Figures 3–6 compare MACE and bleeding risks among different subgroups of patients receiving OAC monotherapy vs. those receiving combination therapy. Similar to the aforementioned findings, the use of combination therapy was associated with significantly higher risks of ischemic stroke, AMI, all-cause mortality, and hemorrhagic stroke than OAC monotherapy in all subgroups except for age; however, patients aged ≥65 years had higher risks of all-cause mortality (*P* for interaction = 0.029) and hemorrhagic stroke (*P* for interaction = 0.011).

### MACE and Bleeding Risks Associated With Warfarin Alone or in Combination With Antiplatelets

As shown in Table 3, we divided the matched cohort into four groups in terms with OACs: DOAC monotherapy, DOAC plus antiplatelets, warfarin monotherapy, and warfarin plus antiplatelets. We found that warfarin was associated with higher risks of all-cause mortality (warfarin monotherapy: HR 1.90, 95% CI 1.12–3.22, *P* = 0.018; warfarin plus antiplatelets: HR 2.33, 95% CI 1.47–3.69, *P* < 0.001) compared with DOAC monotherapy. Furthermore, we found that the combination of warfarin with antiplatelets was associated with increased risks of ischemic stroke (HR 2.31, 95% CI 1.47–3.62, *P* < 0.001), AMI (HR 9.4, 95% CI 2.31–38.32, *P* = 0.002), and GI bleeding (HR 2.35, 95% CI 1.38–4.00, *P* = 0.002) among the four subgroups. The risk of CKD progression did not differ between those receiving DOACs and those receiving warfarin, irrespective of whether they were used in combination with antiplatelets.

**Table 3:** Risks of all-cause mortality, progression of chronic kidney disease, adverse cardiovascular events and bleeding complications between the four groups of antithrombotic treatments in patients with chronic kidney disease with atrial fibrillation and coronary artery disease.

**Outcomes**	DOAC^*^ monotherapy	**Warfarin**		DOAC^*^ **plus**		**Warfarin plus**	
		**monotherapy**		**antiplatelets**		**antiplatelets**	
	**Crude HR**	**Crude HR**	***P*-value**	**Crude HR**	***P*-value**	**Crude HR**	***P*-value**
	**(95% CI)**	**(95% CI)**		**(95% CI)**		**(95% CI)**	
**All-cause mortality**	Reference	1.90 (1.12–3.22)	0.018	1.45 (0.9–2.33)	0.123	2.33 (1.47–3.69)	<0.001
**Progression of CKD**							
eGFR decline > 20%	Reference	1.5 (0.91–2.48)	0.113	1.05 (0.69–1.58)	0.828	1.09 (0.72–1.64)	0.677
eGFR decline > 30%	Reference	1.41 (0.64–3.1)	0.387	1.22 (0.65–2.27)	0.541	1.51 (0.81–2.79)	0.195
eGFR decline > 40%	Reference	0.85 (0.3–2.39)	0.759	0.81 (0.38–1.73)	0.594	1.22 (0.59–2.52)	0.598
eGFR decline > 50%	Reference	1.24 (0.31–4.97)	0.760	0.67 (0.21–2.12)	0.492	1.25 (0.42–3.7)	0.686
End stage renal disease^†^	Reference	3.53 (0.37–34.01)	0.274	0.79 (0.08–7.59)	0.838	6.93 (0.94–51.11)	0.058
Composite renal outcomes^‡^	Reference	1.15 (0.23–5.71)	0.864	0.25 (0.05–1.26)	0.093	1.36 (0.4–4.67)	0.626
**Major adverse cardiac events**							
Ischemic stroke	Reference	1.03 (0.56–1.9)	0.922	2.52 (1.6–3.95)	<0.001	2.31 (1.47–3.62)	<0.001
AMI	Reference	1.54 (0.26–9.23)	0.636	5.96 (1.45–24.58)	0.013	9.4 (2.31–38.32)	0.002
Systemic embolism	Reference	2.45 (0.95–6.35)	0.064	1.94 (0.83–4.55)	0.127	2.11 (0.91–4.91)	0.084
TIA	Reference	0.33 (0.09–1.27)	0.108	1.12 (0.52–2.41)	0.780	0.66 (0.29–1.47)	0.307
PAOD	Reference	0.75 (0.15–3.84)	0.728	0.58 (0.15–2.25)	0.434	0.41 (0.10–1.63)	0.204
Hospitalization for CHF	Reference	1.12 (0.87–1.44)	0.391	0.89 (0.73–1.1)	0.285	1.19 (0.98–1.45)	0.083
**Bleeding complications**							
Hemorrhagic stroke	Reference	0.79 (0.11–5.63)	0.813	3.31 (0.79–13.91)	0.102	3.03 (0.73–12.68)	0.128
GI bleeding	Reference	2.28 (1.23–4.22)	0.009	1.28 (0.74–2.24)	0.375	2.35 (1.38–4.00)	0.002
Other bleeding	Reference	0.84 (0.50–1.39)	0.489	1.1 (0.74–1.63)	0.631	0.75 (0.5–1.12)	0.161

* *Refers to apixaban, dabigatran, rivaroxaban, and edoxaban*.

† *Defined as eGFR of <15 mL/min per 1.73 m^2^, necessitating long-term dialysis*.

‡ *eGFR decline of 50% from baseline or eGFR <15 mL/min per 1.73 m^2^, necessitating long-term dialysis*.

*DOAC, direct oral anticoagulant; HR, hazard ratio; CI, confidence interval; CKD, chronic kidney disease; eGFR, estimated glomerular filtration rates; ESRD, end-stage renal disease; TIA, transient ischemic attack; PAOD, peripheral artery occlusive disease; AMI, acute myocardial infarction, CHF, congestive heart failure; GI, gastrointestinal*.

### Risks of Thromboembolism and Bleeding Between Warfarin and DOACs

**Figure 3:**
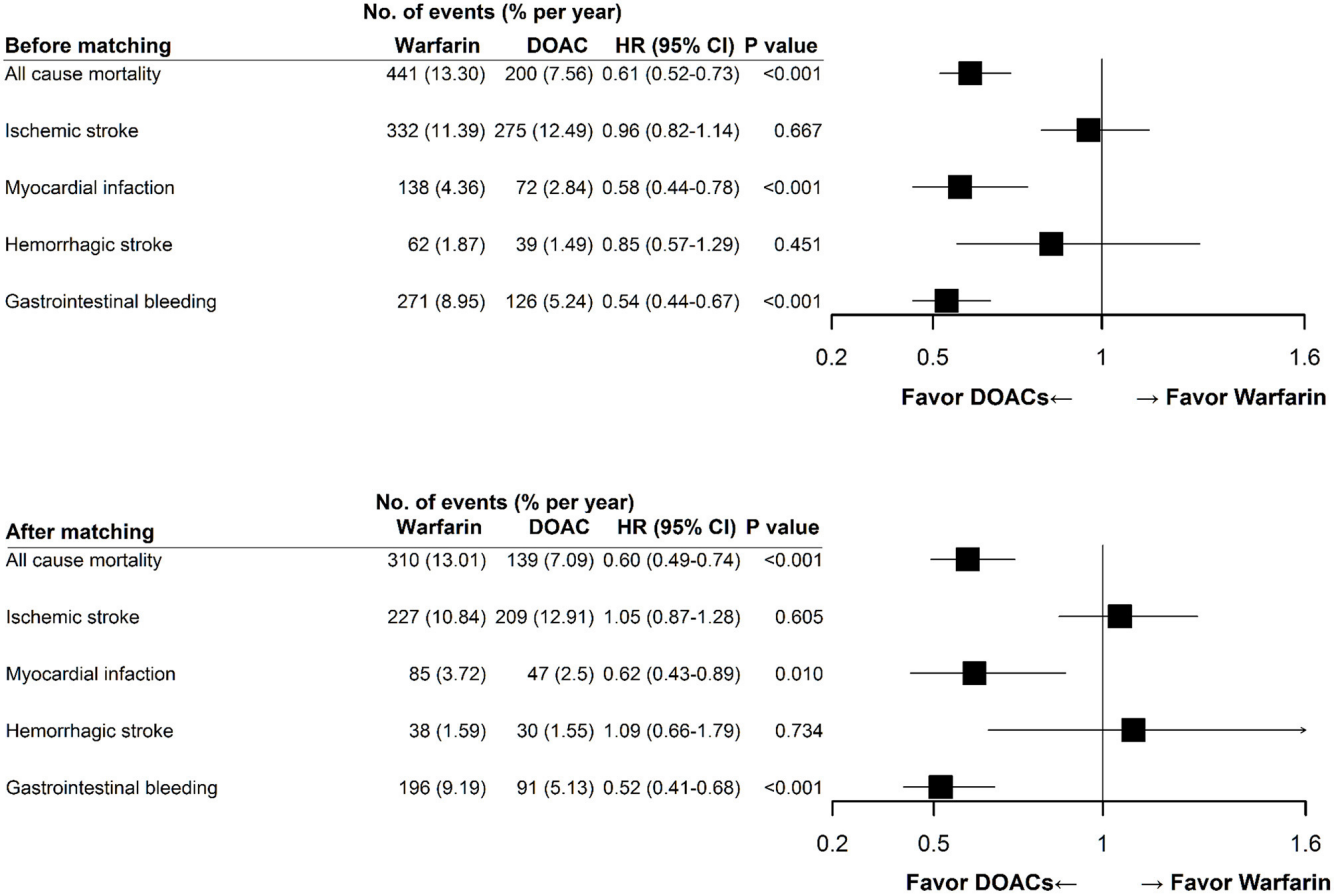
Clinical endpoints of direct oral anticoagulant use compared with warfarin use. Multivariable Cox proportional hazards model showed that DOAC use was associated with lower rates of all-cause mortality, myocardial infarction, and gastrointestinal bleeding, and a similar rate of ischemic and hemorrhagic stroke compared with warfarin use. These findings were consistent before and after propensity score matching. DOAC, direct oral anticoagulant; HR, hazard ratio; CI, confidence interval.

Figure 3 illustrates the risks of thromboembolism and major bleeding associated with warfarin compared with DOACs. In the matched CKD cohort, the risks of all-cause mortality (HR 0.60, 95% CI 0.49–0.74, *P* < 0.001), AMI (HR 0.62, 95% CI 0.43–0.89, *P* = 0.013), and GI bleeding (HR 0.52, 95% CI 0.41–0.68, *P* < 0.001) were significantly lower in patients treated with DOACs compared with those treated with warfarin. Nevertheless, the risks of ischemic and hemorrhage stroke were not different between the two groups. These findings were consistent before and after propensity score matching.

## Discussion

This large-scale retrospective cohort study showed that OAC monotherapy appears to a preferable antithrombotic therapy in patients with CKD with concomitant AF and CAD who had not undergone PCI. The findings of propensity score-matched analysis revealed that the additional use of antiplatelets along with OACs did not exert a stronger protective effect on ischemic stroke and AMI but significantly increased the risk of hemorrhagic stroke. The results were consistent across subgroups categorized by sex, history of hypertension or DM, and baseline eGFR. We also found that the use of DOACs in patients with CKD was associated with a lower risk of all-cause mortality, AMI, and GI bleeding than the use of warfarin. Our study indicated that DOAC monotherapy might be feasible for the management of concurrent AF and CAD in high-risk CKD patients.

To the best of our knowledge, this is the first study to show that OAC alone significantly reduced the CV risks and resulted in lower bleeding and mortality risks compared with combination therapy among patients with CKD and concomitant AF and CAD. Antiplatelets have been considered the drug of choice on primary and secondary preventions in patients with CAD and CKD (6), whereas OAC therapy is crucial for lowering the risk for stroke and thromboembolism in patients with concomitant AF and CKD (7). Our finding has clinical relevance since some physicians prefer prescribing antiplatelets instead of OACs for CKD patients at high bleeding risk. However, irrespective of the antiplatelet regimen used, the deletion of OAC from treatment is inadequate for the prevention of thromboembolism in patients with CKD with a CHA_2_DS_2_-VASc score of 2 or higher (8). On the other hand, we observed that the combined use of antiplatelets with OACs might enhance the risk of AMI among patients with CKD compared to use OACs alone. Although the use of antiplatelets can reduce the risk of ischemic stroke and acute coronary syndrome in patients with normal kidney function, some studies have reported the phenomenon of “antiplatelet resistance” characterized by a poor response to aspirin or clopidogrel in patients with CKD (9, 10). Besides, Jeong et al. have reported that the suboptimal response to antiplatelets is nearly 70% in some of the Asian communities due to genetic polymorphism, and suggested a different therapeutic window of platelet reactivity in East Asians (11, 12). The failure to suppress platelet activity leads to the increased thrombogenicity and may explain the higher risks of ischemic stroke and AMI despite using combination therapy among our participants. Therefore, we suggested that an appropriate OAC use is critical to overcoming the low effectiveness of antiplatelets in patients with CKD with concomitant AF and CAD, especially in those for whom PCI for coronary revascularization is not suitable.

Previous studies and a recent meta-analysis have reached the same conclusion to use OAC monotherapy in patients with AF with stable CAD for 1 year or more after PCI (13, 14). Nevertheless, patients with CKD were often excluded from such clinical trials. By contrast, we included patients with CKD with concomitant CAD who had not yet received PCI. Large-scale prospective CKD trials evaluating the relevance of the proposed treatment strategy are lacking. Our study is the first to illustrate the suitability of OAC prescription in patients with CKD with concomitant AF and CAD.

Although warfarin is the standard treatment for patients with CKD stage 4–5D, we found that DOACs can be a preferred option for the prevention of thromboembolism irrespective of the levels of eGFR. Besides, some observational studies have shown that the renal function of warfarin users tends to decline faster than that of DOACs (15, 16). To explain this phenomenon, Brodsky et al. reported that the nephrotoxicity of warfarin were positively correlated with excessive anticoagulation with an international normalized ratio (INR) of >3.0, which may cause clinically relevant bleeding or glomerular hemorrhage termed “anticoagulant-related nephropathy” (17). In this study, we did not find that DOACs were superior to warfarin in terms of renal outcomes; however, DOACs can be an appropriate and safe antithrombotic treatment for patients with CKD, even in the pre-dialysis stages. This finding was parallel to recent studies that suggested DOACs had comparable efficacy and were safer than warfarin in patients with an eGFR of <15 mL/min per 1.73 m^2^ and those on hemodialysis (HD) (18–21). Recently the Kidney Disease: Improving Global Outcomes (KDIGO) conference approved the consideration regarding the use of a lower dose of apixaban or rivaroxaban in patients with advanced CKD (22). Accordingly, DOACs potentially have equal effectiveness as warfarin and better safety outcomes in patients with AF with advanced CKD.

A substantial proportion of CKD patients inevitably develop ESRD, leading to high CV risk attributed to anemia, endothelial dysfunction, vascular calcification, and oxidative stress (23). Dialysis therapy *per se* significantly impacts coagulation and thrombosis, and warfarin has also been associated with vascular calcification in HD patients (24–26). Randhawa et al. conducted a meta-analysis with 15 observational studies reporting the outcomes of 47,480 patients with AF and ESRD. They found the use of warfarin had no benefits on lowering risks for ischemic stroke, major bleeding, and mortality, but with a significantly higher risk of hemorrhagic stroke in ESRD patients (27). A recent network meta-analysis also showed no evidence of reducing thromboembolic events with warfarin or DOACs in patients with AF and ESRD (28). Although an increasingly popular approach is to use apixaban for stroke prevention among patients with AF and ESRD, the outcomes derived from previous studies were inconstant (18, 20, 29, 30). Therefore, the lack of robust evidence of an appropriate OAC therapeutic approach for dialysis patients highlights the urgent need for additional research in this population.

This study has some limitations. First, the study population was composed of Taiwanese patients from a single center; thus, the results may not be applicable to other ethnic groups. Second, because of the lack of specific details regarding the time in the therapeutic range (6) of warfarin, we could not assess the adequacy of OAC therapy in patients treated with warfarin. In consideration of bleeding tendency attributed to uremic toxins, anemia, and platelet dysfunction in patients with advanced CKD and that undergoing dialysis, the INR target of warfarin therapy in patients with ESRD was apparently conservative in clinical practice. We estimated a low TTR in warfarin users, which reflects poor anticoagulation control and might have affected outcomes in this study. Third, selection bias may exist in a retrospective cohort design. However, we adjusted for potential confounders by using propensity score matching. Moreover, we have to acknowledge that the distributions of CKD among our participants were mainly in stages 3 and 4, whereas the number of ESRD patients taking OACs is small; thereby, our findings might not apply to dialysis-dependent CKD patients. Finally, our study is observational in nature; thus, it cannot prove causality. Nevertheless, given the limited evidence from RCTs on antithrombotic therapy exclusively for patients with CKD, our results indicate the effectiveness and safety of using DOACs alone for AF and CAD among patients with CKD in routine clinical practice.

## Conclusions

In patients with CKD with concomitant AF and CAD who had not undergone PCI, OAC in combination with antiplatelets might not provide additional benefits for the prevention of MACE and further be associated with a higher risk of bleeding events. DOACs can be the preferred OAC therapy than warfarin, with a lower risk of all-cause mortality as well as AMI and GI bleeding. Additional prospective clinical studies are needed to reinforce our findings.

## Data Availability Statement

The raw data supporting the conclusions of this article will be made available by the authors, without undue reservation.

## Ethics Statement

The studies involving human participants were reviewed and approved by Institutional Review Board of the Taipei Veterans General Hospital. Written informed consent for participation was not required for this study in accordance with the national legislation and the institutional requirements.

## Author Contributions

Y-PL and D-CT were involved in planning and supervised the work. K-HL and S-MO processed the experimental data, performed the analysis, drafted the manuscript, and designed the figures. Y-CC designed the model and the computational framework. M-TT aided in interpreting the results and worked on the manuscript. All authors discussed the results and commented on the manuscript.

## Funding

This work was supported by grants from the Ministry of Science and Technology, ROC (109-2314-B-010-056-MY3 and 109-2314-B-075-066) and Taipei Veterans General Hospital (V108D42-004-MY3-2, V109D50-001-MY3-1, V108C-103, V109C-114, and V109B-016). This work was also financially supported by the Center for Intelligent Drug Systems and Smart Bio-devices (IDS^2^B) from the Featured Areas Research Center Program within the framework of the Higher Education Sprout Project by the Ministry of Education (MOE) in Taiwan, and Foundation for Poison Control (FPC-110-003).

## Conflict of Interest

The authors declare that the research was conducted in the absence of any commercial or financial relationships that could be construed as a potential conflict of interest.

## Publisher's Note

All claims expressed in this article are solely those of the authors and do not necessarily represent those of their affiliated organizations, or those of the publisher, the editors and the reviewers. Any product that may be evaluated in this article, or claim that may be made by its manufacturer, is not guaranteed or endorsed by the publisher.
