# Supplementary material for: Antithrombotic Therapy for Chronic Kidney Disease Patients With Concomitant Atrial Fibrillation and Coronary Artery Disease
Source: Front Cardiovasc Med. 2021 Oct 8;8:751359. doi: 10.3389/fcvm.2021.751359 (PMC8531471; doi:10.3389/fcvm.2021.751359)

Supplemental Material

Antithrombotic therapy for chronic kidney disease patients with concomitant atrial fibrillation and coronary artery disease

Running title: Antithrombotics in CKD+ AF+ CAD

Kuo-Hua Lee, MD, [a,b,c*] Shuo-Ming Ou, MD, [a,b,c*] Yuan-Chia Chu, PhD, [d,e] Yao-Ping Lin, MD, PhD,[a,b,c] Ming-Tsun Tsai, MD,[a,b,c] and Der-Cherng Tarng, MD, PhD,[a,b,c,f] Evaluating the Prognosis and Impacts in CKD (EPIC) Taiwan Research Group

[a] Division of Nephrology, Department of Medicine, Taipei Veterans General Hospital, Taipei City, Taiwan

[b] Institute of Clinical Medicine, National Yang-Ming University, Taipei City, Taiwan

[c] Center for Intelligent Drug Systems and Smart Bio-devices (IDS^{2}B), Hsinchu, Taiwan

[d] Information Management Office, Taipei Veterans General Hospital, Taipei City, Taiwan

[e] Big Data Center, Taipei Veterans General Hospital, Taipei City, Taiwan

[f] Department and Institute of Physiology, National Yang-Ming University, Taipei City, Taiwan

*Drs Kuo-Hua Lee and Shuo-Ming Ou contributed equally to this study.

Correspondence:

Der-Cherng Tarng, MD, PhD

Division of Nephrology, Department of Medicine, Taipei Veterans General Hospital 201, Section 2, Shih-Pai Road, Taipei 11217, Taiwan

Phone: 886-2-28757517

Fax: 886-2-28757841

Email: dctarng@vghtpe.gov.tw

Table of Contents

**Supplement Tables**	
Supplement Table 1. Propensity Score Model Results of Probability of Oral Anticoagulant Monotherapy	3
**Supplement Figures**	
Supplement Figure 1. Distributional Balance for Propensity Score Before and After Matching	5
Supplement Figure 2. Covariate Balance Before and After Propensity Score Matching	6
Supplement Figure 3. Subgroup analysis of Risk of Ischemic Stroke Among Oral Anticoagulant Monotherapy Group and Combination Therapy Group	7
Supplement Figure 4. Subgroup analysis of Risk of Acute Myocardial Infarction Among Oral Anticoagulant Monotherapy Group and Combination Therapy Group	8
Supplement Figure 5. Subgroup analysis of Risk of All-cause Mortality Among Oral Anticoagulant Monotherapy Group and Combination Therapy Group	9
Supplement Figure 6. Subgroup analysis of Risk of Hemorrhagic Stroke Among Oral Anticoagulant Monotherapy Group and Combination Therapy Group	10

Supplement Table 1. Propensity Score Model Results of Probability of OAC monotherapy

			95% CI		
**Parameter**	**Estimate**	**Odds Ratios**	**Lower**	**Upper**	**P value**
**Age, years**	-0.012	0.988	-0.022	-0.002	0.016
**Male**	-0.357	0.700	-0.562	-0.151	0.001
**Cholesterol, mg/dL**	0.003	1.003	-0.001	0.007	0.199
**HbA1c, %**	-0.001	0.999	-0.004	0.002	0.519
**Hemogloblin**	-0.041	0.960	-0.095	0.014	0.144
**eGFR, mL/min/1.73 m2**					
**> 90**					
**60–89**	0.001	1.001	-0.429	0.430	0.997
**30–59**	-0.072	0.930	-0.517	0.373	0.751
**15-29**	-0.145	0.865	-0.687	0.397	0.599
**<15**	-0.190	0.827	-0.804	0.423	0.543
**TG, mg/dL**	0.001	1.001	-0.001	0.003	0.199
**LDL, mg/dL**	-0.002	0.998	-0.007	0.003	0.531
**Glucose, mg/dL**	0.001	1.001	0.000	0.003	0.105
**UPCR, mg/mg**	-0.002	0.998	-0.010	0.006	0.634
**Hypertension**	-0.299	0.741	-0.521	-0.078	0.008
**DM**	-0.051	0.951	-0.295	0.194	0.685
**CHF**	0.046	1.047	-0.151	0.244	0.647
**Malignancy**	-0.116	0.890	-0.347	0.115	0.326
**ACEIs/ARBs**	-0.187	0.830	-0.399	0.025	0.084
**β-blockers**	-0.217	0.805	-0.427	-0.007	0.043
**α-blockers**	-0.293	0.746	-0.525	-0.060	0.014
**CCBs**	-0.013	0.988	-0.221	0.196	0.906

**Statins**	-0.738	0.478	-0.965	-0.512	<0.001
**OHAs**	-0.114	0.892	-0.422	0.194	0.467
**Insulins**	-0.166	0.847	-0.430	0.099	0.220

Abbreviations: OAC, oral anticoagulant; SMD, standardized mean difference; LDL, low-density lipoprotein; TG, triglyceride; HbA1C, glycated hemoglobin; eGFR, estimated glomerular filtration rate; UPCR, urine protein-to-creatinine ratio; DM, diabetes mellitus; CHF, congestive heart failure; ACEI, angiotensin converting enzyme inhibitor; ARB, angiotensin receptor blocker; CCB, calcium channel blockers; OHA, oral hypoglycemic agent

Supplement Figure 1. Distributional Balance for Propensity Score Before and After Matching

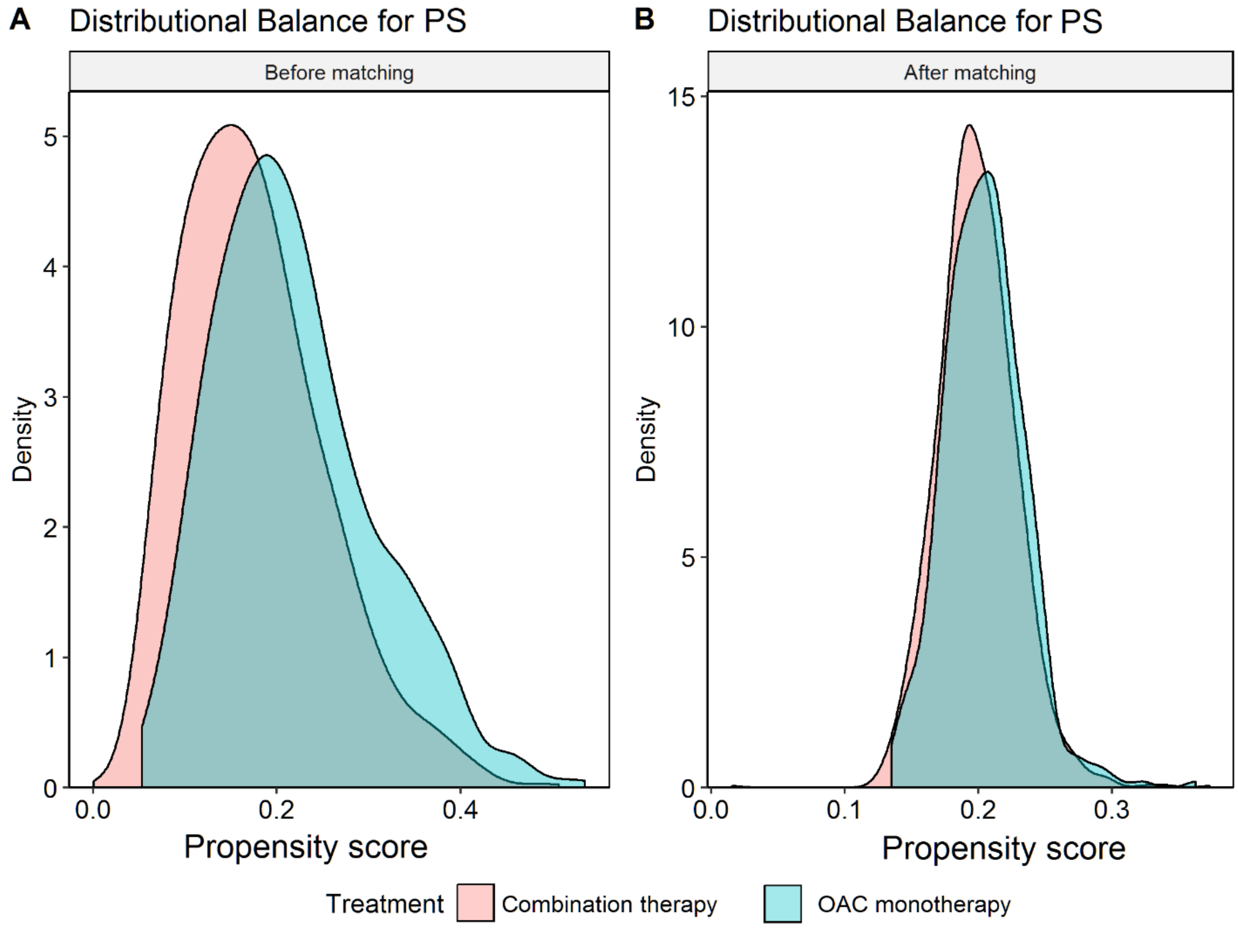

Supplement Figure 2. Covariate Balance Before and After Propensity Score Matching

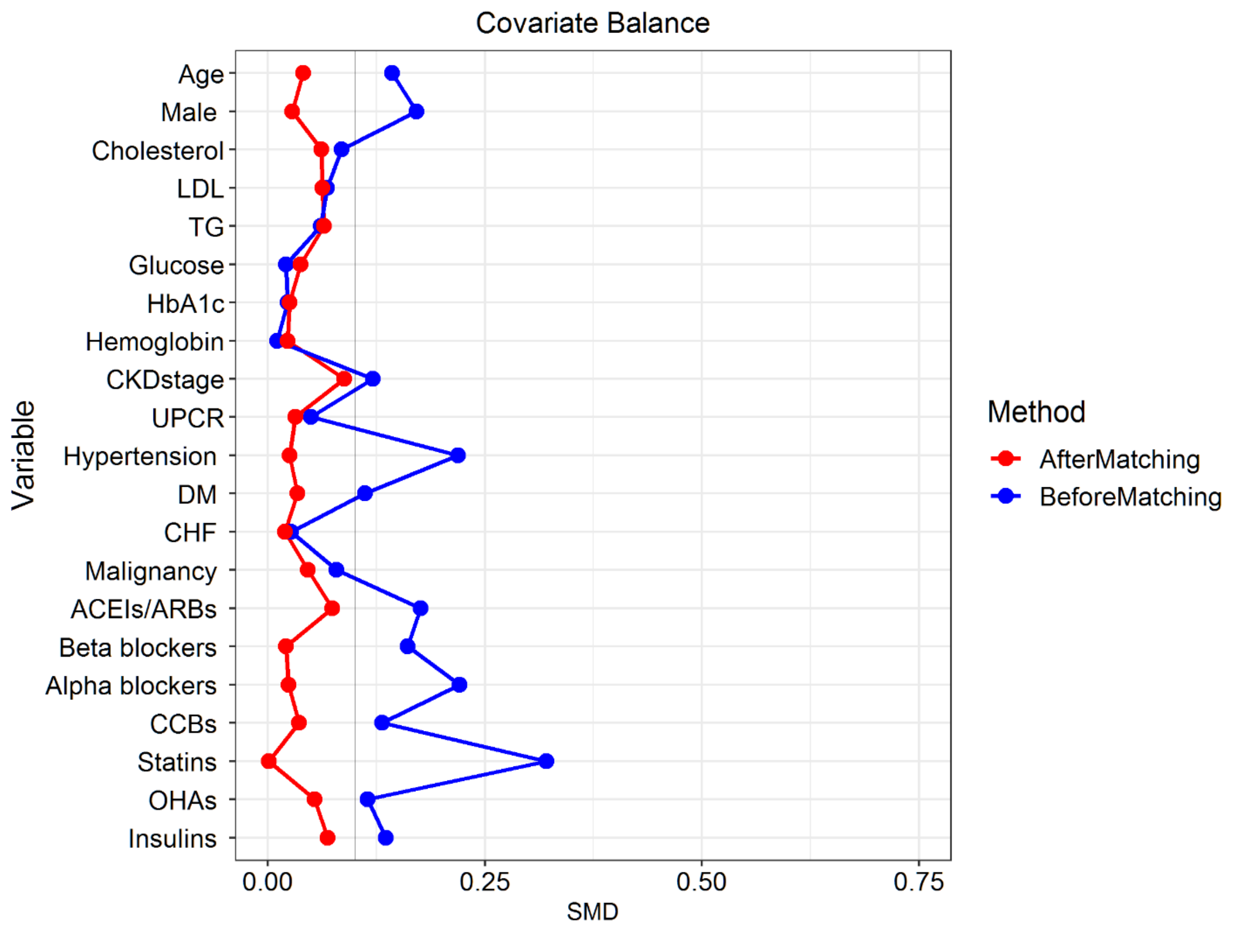

Supplement Figure 3. Subgroup analysis of Risk of Ischemic Stroke Among Oral Anticoagulant Monotherapy Group and Combination Therapy Group

Subgroup	Hazard Ratio(95% CI)	P for interaction
**All patients**	**2.37 (1.72-3.27)**	
**Age**		**0.291**
< 65 years	1.44 (0.56-3.73)	
≥ 65 years	2.49 (1.77-3.51)	
**Sex**		**0.070**
Female	4.10 (2.01-8.37)	
Male	1.96 (1.36-2.81)	
**Hypertension**		**0.594**
No	2.86 (1.32-6.20)	
Yes	2.27 (1.59-3.23)	
**Diabetes mellitus**		**0.824**
No	2.43 (1.62-3.64)	
Yes	2.24 (1.32-3.81)	
**Estimated glomerular filtration rate**		**0.344**
≥ 45 ml/min/1.73m2	2.92 (1.54-5.56)	
< 45 ml/min/1.73m2	2.12 (1.46-3.08)	
**UPCR**		**0.080**
< 1000 mg/g	2.02 (1.42-2.86)	
> 1000 mg/g	4.50 (1.97-10.27)	
**HAS-BLED score**		**0.986**
< 2	1.66 (0.32-8.62)	
≥ 2	2.21 (1.59-3.07)	

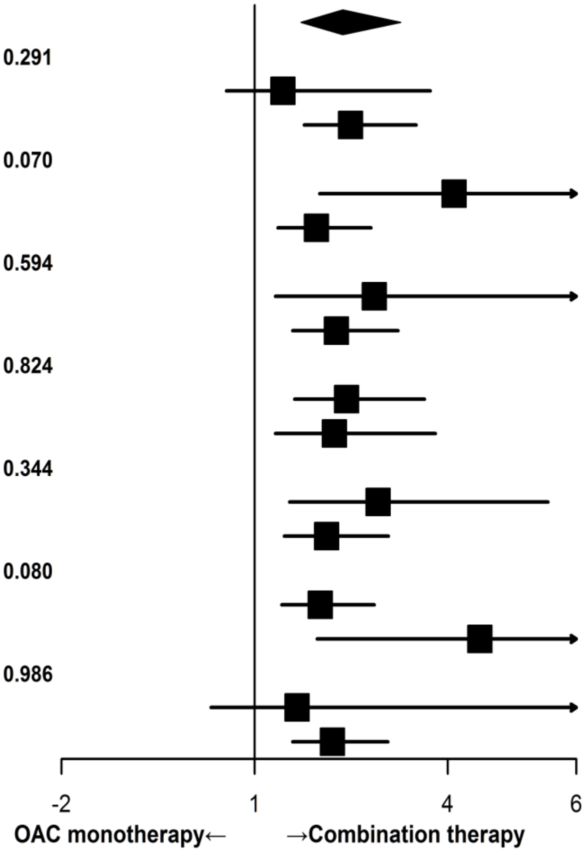

Supplement Figure 4. Subgroup analysis of Risk of Acute Myocardial Infarction Among Oral Anticoagulant Monotherapy Group and Combination Therapy Group

Subgroup	Hazard Ratio(95% CI)	P for interaction
**All patients**	**6.14 (2.51-15.00)**	
**Age**		**0.732**
< 65 years	4.45 (0.59-33.35)	
≥ 65 years	6.54 (2.41-17.75)	
**Sex**		**0.904**
Female	5.82 (1.41-23.99)	
Male	6.43 (2.03-20.35)	
**Hypertension**		**0.712**
No	5.12 (1.24-21.18)	
Yes	6.90 (2.18-21.81)	
**Diabetes mellitus**		**0.28**
No	9.93 (2.44-40.43)	
Yes	3.57 (1.11-11.47)	
**Estimated glomerular filtration rate**		**0.276**
≥ 45 ml/min/1.73m2	3.58 (1.12-11.50)	
< 45 ml/min/1.73m2	9.71 (2.38-39.52)	
**UPCR**		**0.509**
< 1000 mg/g	7.57 (2.40-23.91)	
≥ 1000 mg/g	3.89 (0.93-16.29)	
**HAS-BLED score**		**0.882**
< 2	6.19 (1.45-26.41)	
≥ 2	5.82 (1.85-18.34)	

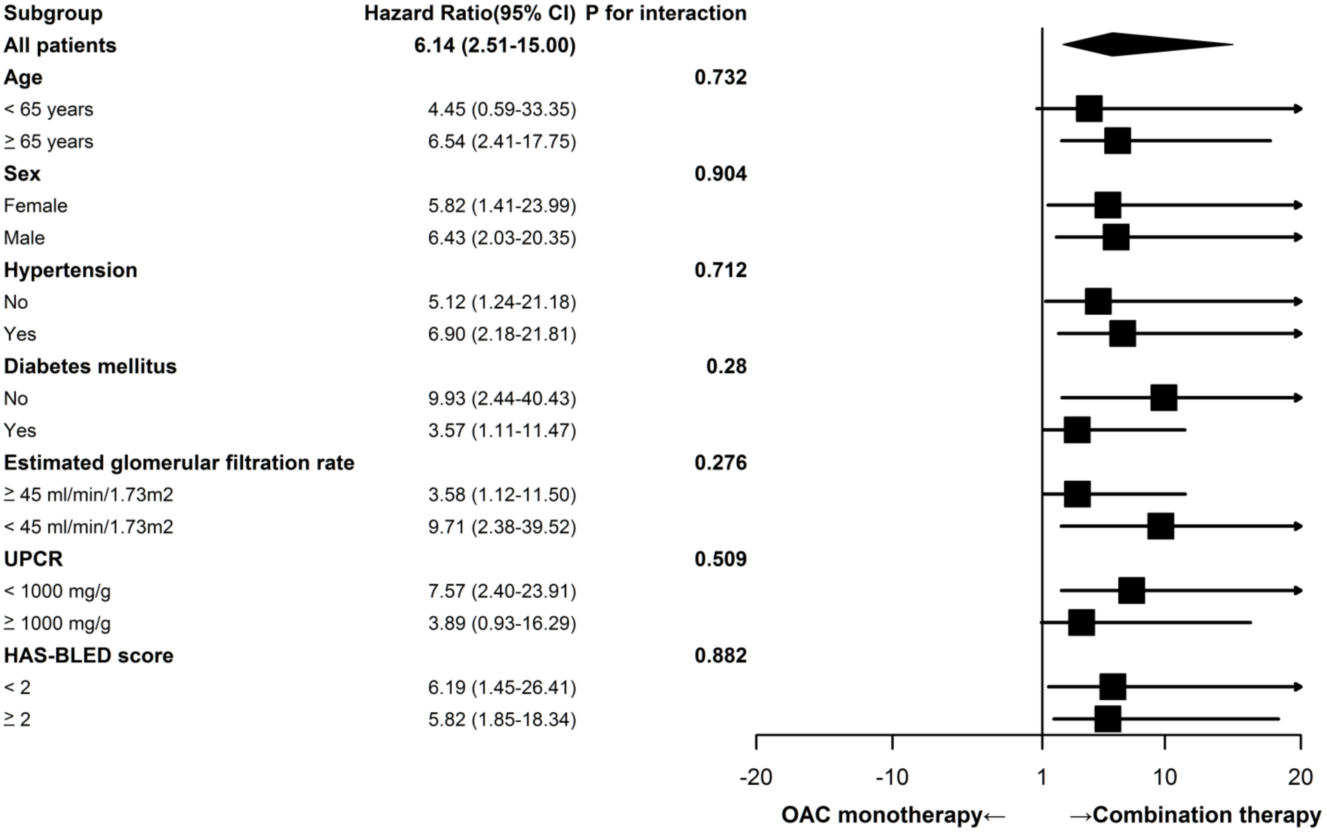

Supplement Figure 5. Subgroup analysis of Risk of All-cause Mortality Among Oral Anticoagulant Monotherapy Group and Combination Therapy Group

Subgroup	Hazard Ratio(95% CI)	P for interaction
**All patients**	**1.31 (1.01-1.71)**	
**Age**		**0.029**
< 65 years	0.49 (0.20-1.21)	
≥ 65 years	1.41 (1.07-1.86)	
**Sex**		**0.225**
Female	1.76 (1.01-3.07)	
Male	1.19 (0.88-1.60)	
**Hypertension**		**0.518**
No	1.13 (0.67-1.91)	
Yes	1.38 (1.02-1.87)	
**Diabetes mellitus**		**0.641**
No	1.25 (0.91-1.72)	
Yes	1.45 (0.90-2.35)	
**Estimated glomerular filtration rate**		**0.713**
≥ 45 ml/min/1.73m2	1.23 (0.80-1.89)	
< 45 ml/min/1.73m2	1.30 (0.93-1.81)	
**UPCR**		**0.762**
< 1000 mg/g	1.35 (0.99-1.84)	
≥ 1000 mg/g	1.19 (0.72-1.98)	
**HAS-BLED score**		**0.640**
< 2	1.02 (0.60-1.73)	
≥ 2	1.17 (0.85-1.60)	

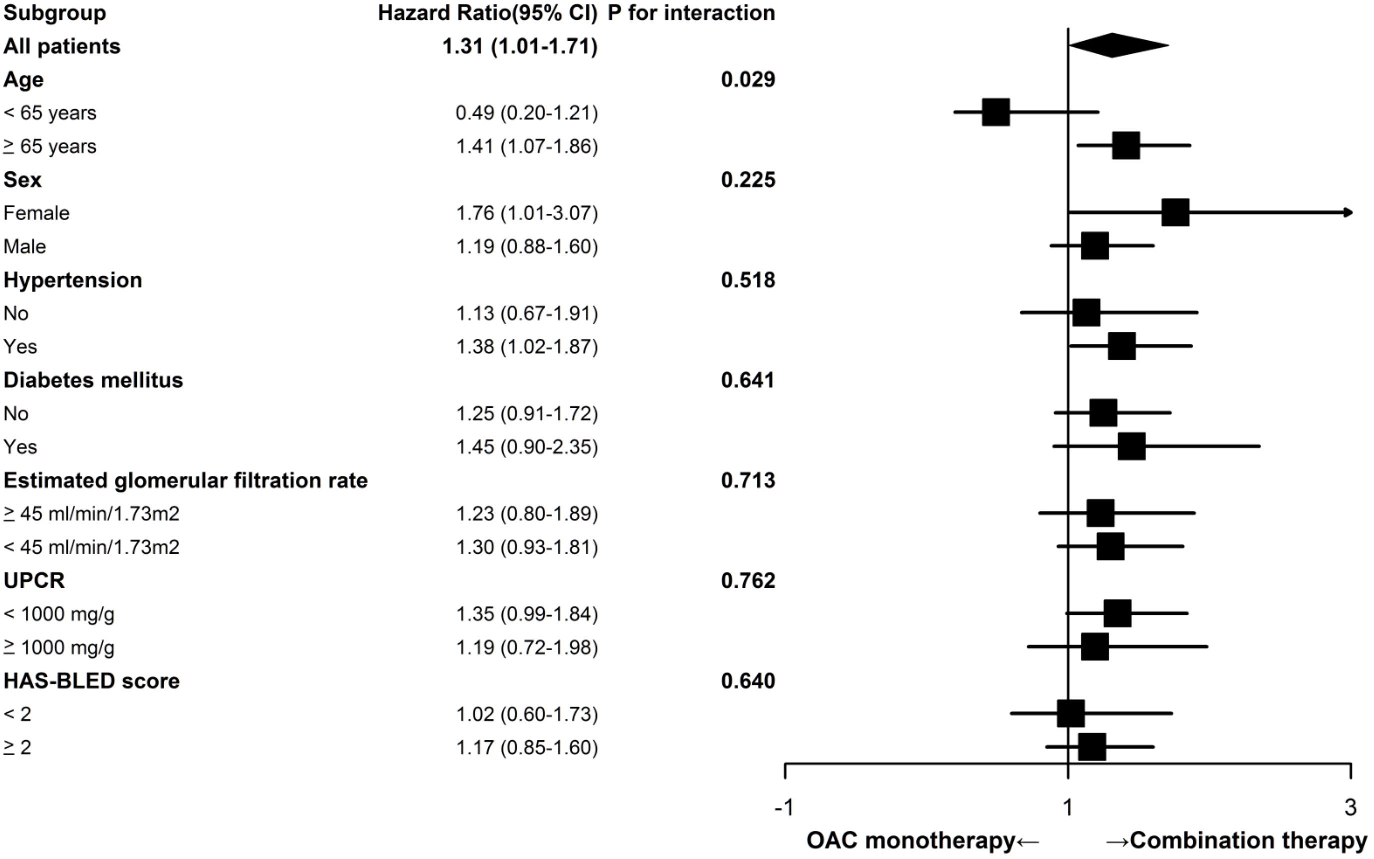

Supplement Figure 6. Subgroup analysis of Risk of Hemorrhagic Stroke Among Oral Anticoagulant Monotherapy Group and Combination Therapy Group

Subgroup	Hazard Ratio(95% CI)	P for interaction
**All patients**	**2.39 (1.25-4.58)**	
**Age**		**0.011**
< 65 years	0.38 (0.09-1.58)	
≥ 65 years	3.25 (1.51-7.00)	
**Sex**		**0.448**
Female	3.37 (1.05-10.88)	
Male	1.96 (0.90-4.29)	
**Hypertension**		**0.851**
No	2.66 (0.63-11.29)	
Yes	2.32 (1.12-4.80)	
**Diabetes mellitus**		**0.477**
No	2.91 (1.17-7.26)	
Yes	1.80 (0.71-4.54)	
**Estimated glomerular filtration rate**		**0.135**
≥ 45 ml/min/1.73m2	1.35 (0.57-3.18)	
< 45 ml/min/1.73m2	3.74 (1.36-10.30)	
**UPCR**		**0.899**
< 1000 mg/g	2.47 (1.14-5.35)	
≥ 1000 mg/g	2.28 (0.69-7.52)	
**HAS-BLED score**		**0.440**
< 2	0.49 (0.03-7.85)	
≥ 2	1.80 (0.91-3.56)	

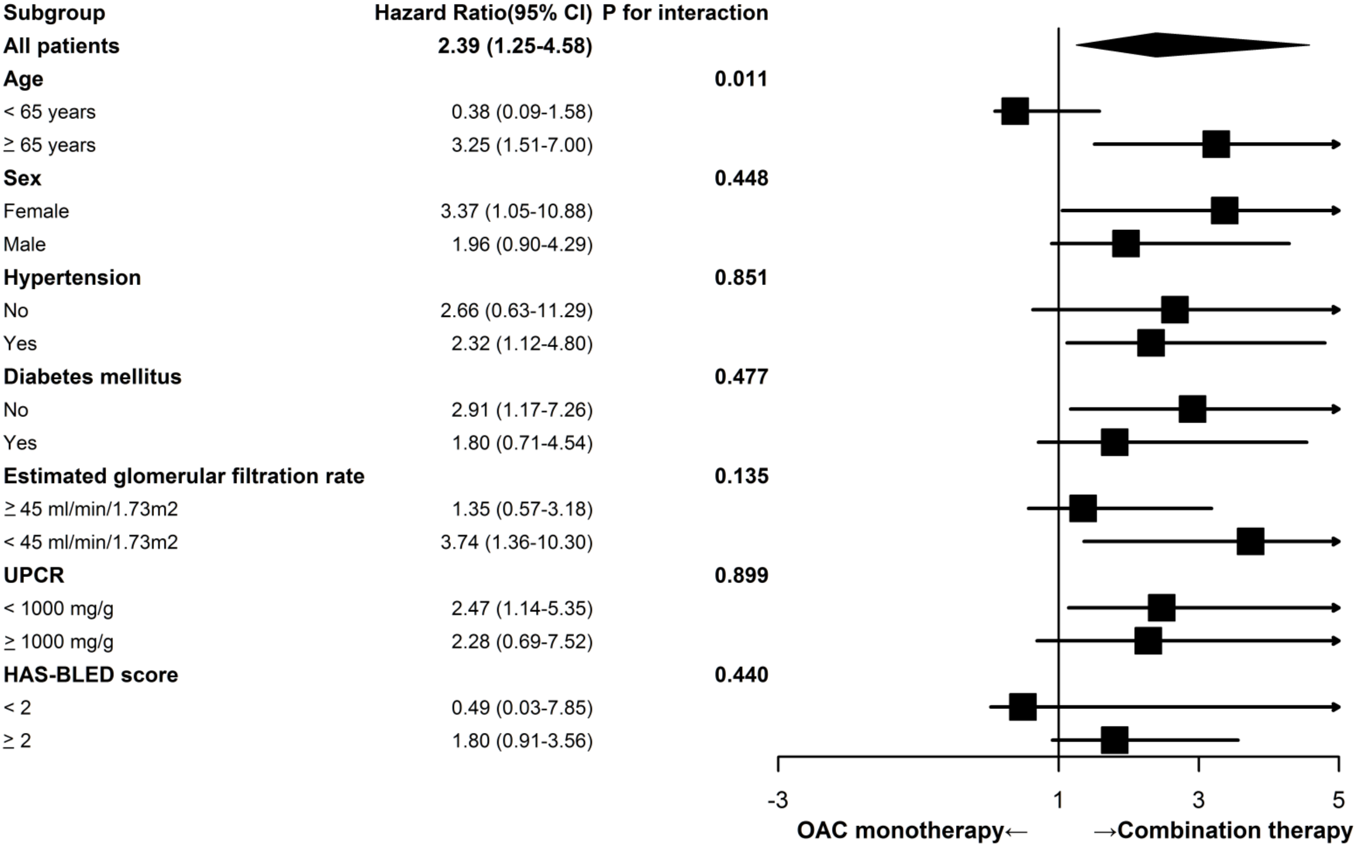

Supplement: Supplementary file 1 [file Data_Sheet_1.PDF]
